# Solamargine induces hepatocellular carcinoma cell apoptosis and autophagy via inhibiting LIF/miR-192-5p/CYR61/Akt signaling pathways and eliciting immunostimulatory tumor microenvironment

**DOI:** 10.1186/s13045-022-01248-w

**Published:** 2022-03-21

**Authors:** Shuangshuang Yin, Wenke Jin, Yuling Qiu, Leilei Fu, Tao Wang, Haiyang Yu

**Affiliations:** 1 Key Laboratory of Pharmacology of Traditional Chinese Medical Formulae, Ministry of Education, and State Key Laboratory of Component-Based Chinese Medicine, Tianjin University of Traditional Chinese Medicine, Tianjin, 301617 China; 2 Sichuan Engineering Research Center for Biomimetic Synthesis of Natural Drugs, School of Life Science and Engineering, Southwest Jiaotong University, Chengdu, 610031 China; 3 School of Pharmacy, Tianjin Medical University, Tianjin, 300070 China

**Keywords:** Hepatocellular carcinoma, Traditional Chinese herb, Solamargine, Apoptosis, Autophagy, Tumor microenvironment

## Abstract

Hepatocellular carcinoma (HCC) is well-known to be a highly prevalent malignant tumor, but the treatment of this pathological state has been still challenging. Solamargine (SM), a traditional Chinese herb-derived compound, has been widely reported to possess multiple antitumor properties. However, whether SM plays a vital role in HCC therapy and how it exerts an antitumor effect remains unclear. Thus, in this study, we demonstrated that SM inhibited the proliferation of HCC and effectively induced HCC cell apoptosis and autophagy in vitro and in vivo. Mechanistically, the oncogenic factor LIF was aberrantly elevated in HCC tissues and down-regulated by SM in HCC cells, as well as subsequently the overexpression of LIF could restore the anti-HCC effects of SM via miR-192-5p/CYR61/Akt signaling pathways. Additionally, SM could repolarize tumor associated macrophages by LIF/p-Stat3 to inhibit the growth and epithelial-mesenchymal transition of HCC, and simultaneously affected other immune cell populations in the immune (tumor) microenvironment by regulating macrophages, such as MDSCs, DCs and T cell populations. Together, these findings exploit the potential use of SM against HCC and shed light on exploring SM as a potent candidate drug for the future HCC therapeutics.

**Supplementary Information:**

The online version contains supplementary material available at 10.1186/s13045-022-01248-w.

**To the Editor,**

Hepatocellular carcinoma (HCC) is well-known to be a malignant cancer and highly effective therapeutic drugs or approaches are insufficiency [1, 2]. Of note, *Solanum nigrum* *L*. has its biological functions of clearing away heat, detoxifying, promoting blood circulation and reducing swelling, and has been widely used in Chinese folk medicine for treating cancers and warts. Solamargine (SM) is a natural compound found in *Solanum nigrum* *L*. with multifaceted antitumor mechanisms [3–5]. However, whether SM plays a vital role in HCC treatment and how it exerts antitumor effect still remains to be discovered.

As expected, SM significantly decreased the viability and proliferation of HCC cells, and increased the apoptotic and autophagic ratio. To ascertain the anti-HCC effects of SM in vivo, a patient-derived tumor xenograft (PDX) mouse model and an orthotopic HCC mouse model were constructed. Tumor growth was significantly slowed, and the deterioration of the liver and lung was ameliorated by SM. SM also promoted apoptosis and autophagy in vivo. (Fig. 1a–c, f and Additional file 1: Figs. S1 and S2). These findings demonstrate that SM markedly inhibits HCC by inducing apoptosis and autophagy in vitro and in vivo.

**Fig. 1:**
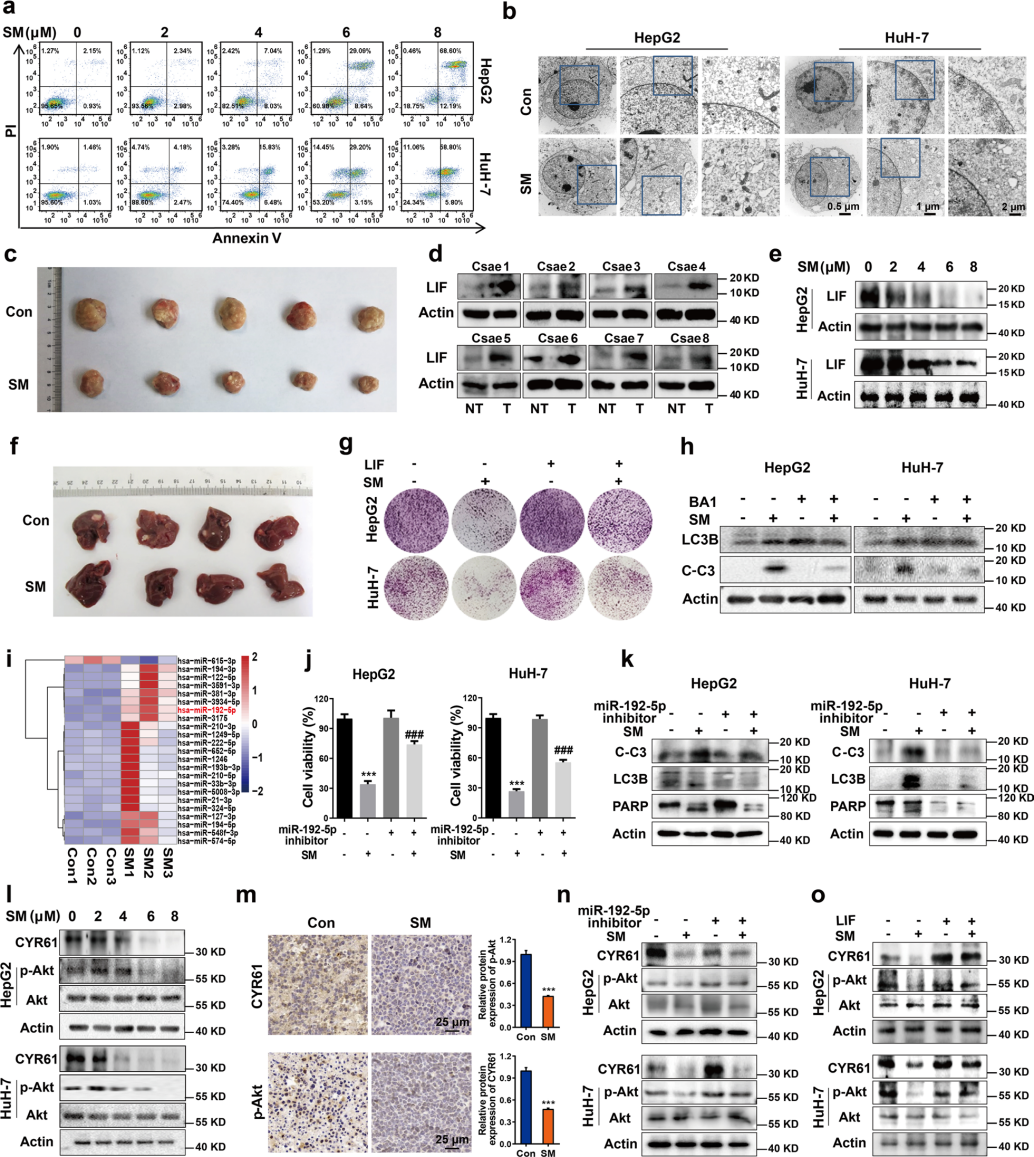
Solamargine induces apoptosis and autophagy by inhibiting LIF/miR-192-5p/CYR61/Akt axis in hepatocellular carcinoma. **a** Representative results of Annexin V-FITC/PI staining of HCC cells treated with SM for 24 h. **b** Autophagy was measured by transmission electron microscopy after treatment with SM in HCC cells. **c** The representative images of isolated tumors derived from PDX mice (*n* = 8). **d** The samples of human HCC patients were collected and the expression of LIF in adjacent or normal tissues was detected by western blot assay. **e** The expression of LIF in HCC cells was examined by western blot following treatment with SM. **f** The representative images of livers derived from orthotopic HCC mice after vehicle and SM treatment (*n* = 10). **g** Colony formation assay of HCC cells treated with SM combined with ectopic LIF or SM alone. **h** The expressions of several key cell apoptosis and autophagy signal regulators were examined by western blotting after treatment with SM combined with BA1 or SM alone. **i** Heatmap of differentially expressed miRNA with significant differences expression in HepG2 cells treated with or without SM (6 µM). **j** HepG2 and HuH-7 cells were treated with SM with or without miR-192-5p inhibitor, and the inhibition of growth was assessed. **k** HCC cells were treated with SM with or without miR-192-5p inhibitor, and the protein expressions of several key cell apoptosis and autophagy were examined by western blotting. **l** The protein expressions of CYR61, p-Akt and total Akt in HCC cells were detected by western blotting. **m** Immunohistochemistry revealed the expressions of p-Akt and CYR61 in tumor tissues of PDX mice. **n–o** HCC cells were treated with SM alone or transfected with miR-192-5p inhibitor (right) or LIF- plasmid (left), the expressions of p-Akt, total Akt and CYR61 were detected by western blotting. Actin was used as a loading control. Data were presented as means ± SD, ns means no significance, *p < 0.05, **p < 0.01, ***p < 0.001; ^#^*p* < 0.05, ^##^*p* < 0.01, ^###^*p* < 0.001

LIF, as a multifunctional cytokine, plays a controversial role in the development of various tumors [6, 7]. RNA-seq analysis was performed to determine the differentially expressed genes in response to SM and found that LIF plays a key regulatory role in the network. Moreover, SM decreased LIF expression both in HCC cells and in the orthotopic HCC mouse, but the tumor cells-inhibitory effect of SM was attenuated by LIF overexpression, and HCC patients with higher expression of LIF had poorer prognoses. (Fig. 1d, e, g and Additional file 1: Figs. S3 and S4a, b). These data indicate that LIF plays a vital role and may be a potential target in SM-mediated inhibition of HCC growth.

The differentially expressed genes regulated by SM were most enriched in apoptosis and autophagy. To explore the relationship between SM-mediated autophagy and apoptosis, bafilomycin A1 (BA1) or siLC3B was used to block autophagy induction. We found that HCC cell viability and the apoptotic rate in response to SM were relieved when combined with BA1 or siLC3B (Fig. 1h and Additional file 1: Fig. S4c–i).

To uncover the potential mechanisms of SM-induced cell death, miRNA-seq analysis was used to examine differentially expressed miRNAs. Among these miRNAs, miR-192-5p loss has been reported to further initiate HCC malignancy [8], and SM treatment markedly upregulated miR-192-5p in HCC cells. Furthermore, a miR-192-5p inhibitor partially blocked SM-induced apoptosis and autophagy, and patients with lower expression levels of miR-192-5p had poorer prognoses. Next, we examined the differentially expressed genes obtained by RNA-seq analysis and found that CYR61, acting as an oncogene [9], was obviously downregulated by SM, and HCC patients with higher expression of CYR61 exhibited poorer survival rates. Besides, inhibition of miR-192-5p expression significantly enhanced CYR61 expression in HCC cells, and genes regulated by SM were most enriched in the PI3K/Akt signaling pathway. Notably, inhibition of miR-192-5p also rescued p-Akt expression. Additionally, the levels of CYR61 and p-Akt were dramatically lower in the SM treatment group in PDX mice. Furthermore, the expression of miR-192-5p, CYR61 and p-Akt were regulated by SM, were effectively inverted by LIF overexpression. Interestingly, the protein–protein interaction (PPI) network of CYR61 has significant overlap with the PPI network of LIF, and the expression of LIF was positively associated with CYR61 and negatively correlated with miR-192-5p in HCC tissues. (Fig. 1i–o and Additional file 1: Fig. S5). These findings demonstrate that SM induces autophagy and apoptosis may via LIF/miR-192-5p/CYR61/Akt axis to hinder HCC development.

In addition to the abovementioned apoptosis and autophagy-modulating mechanisms, SM also significantly repolarized M2 macrophages toward M1-like phenotype to kill tumor cells via phagocytosis^10^ in both THP-1 and RAW 264.7 cells. Moreover, HCC cell invasiveness induced by M2 macrophages was repressed by SM via LIF/p-Stat3 signaling. The role of TAMs was also evaluated in vivo, and we found that SM induced DCs activation or recruitment in tumors but not the spleen, and reduced the proportion of G-MDSCs in both tumor and the spleen but had little effect on M-MDSCs. SM also enhanced the percentage of CD4^+^ T cells but did not increase CD8^+^ T cells (Fig. 2 and Additional file 1: Figs. S6 and S7). However, macrophages deficiency weakened the effect of SM on the immune microenvironment against HCC.

**Fig. 2:**
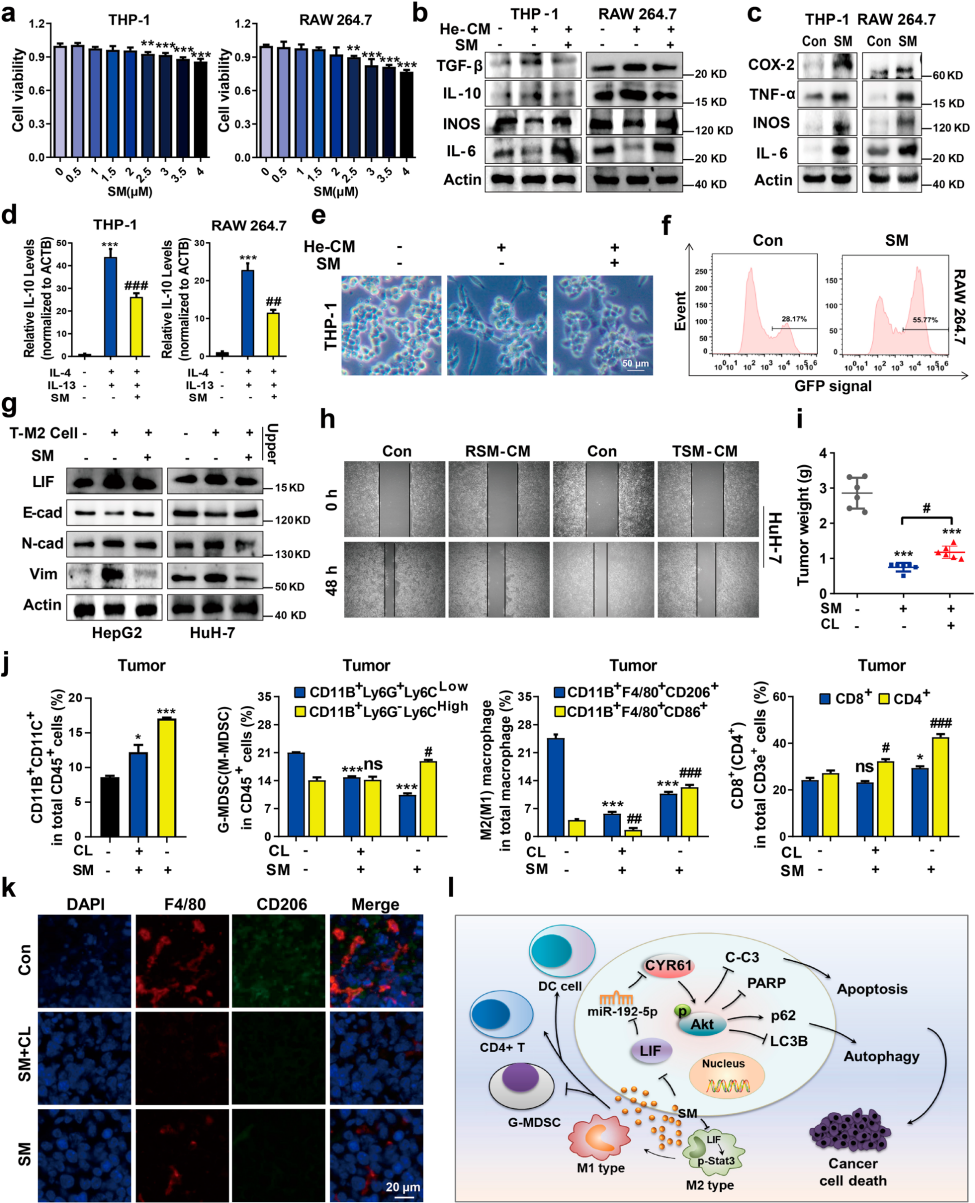
Solamargine elicits an immunostimulatory tumor microenvironment via macrophages in hepatocellular carcinoma. **a** Cell viability was exanimated after SM treatment in THP-1 and RAW 264.7 cells. **b** Co-culture of macrophages and tumor-conditioned medium (TCM) for 24 h to induce M2-like macrophage, then treated with SM for 24 h, the expressions of M2 associated gene (TGF-β, IL-10) and M1 associated gene (INOS, IL-6) were measured. **c** Macrophages were co-cultured with SM for 24 h, the protein expressions of M1 associated genes were measured. **d** Macrophage were co-cultured with IL-4 and IL-13 for 24 h to induce M2-like macrophage, then treated with SM for 24 h, the expressions of M2 associated genes were measured by qRT-PCR assay. **e** The phase-contrast photomicrographs showed the morphology after treatment with SM plus TCM or SM alone. **f** M2-like macrophages were treated with SM for 24 h, the culture medium was aspirated, and macrophages were co-cultured with HuH-7-GFP cells for 12 h. The phagocytosis of macrophages was detected by FACS analysis. **g** M2 macrophage were pretreated with SM and placed in the upper chamber to test the invasion ability of HCC cells in the lower chamber. The expressions of LIF, E-cad, N-cad and Vim were determined by western blotting. **h** HCC cells were treated with or without RSM-CM or TSM-CM. The scratch assay was used to measure migration capabilities of HCC cells. Representative images were shown. **i** The final tumor weight of H22 subcutaneous tumor mice. **j** Tumor-associated macrophages, MDSCs, DCs and infiltrating T cells in endpoint tumors were analyzed by Flow cytometry analysis. **k** Immunofluorescent microscopy images of tissue sections were stained with antibodies against mouse CD206 (green) and F4/80 (red) to observe tumor-associated macrophages. **l** A schematic diagram illustration of dual synergistic anti-cancer activities of SM. Actin was used as a loading control. Data were presented as means ± SD, ns means no significance, *p < 0.05, **p < 0.01, ***p < 0.001; ^#^*p* < 0.05, ^##^*p* < 0.01, ^###^*p* < 0.001

Our results demonstrate that relatively high concentrations of SM may be effective in treating HCC by inducing autophagy and apoptosis via LIF/miR-192-5p/CYR61/Akt axis. Simultaneously, low concentrations of SM exhibits little or no direct toxicity toward macrophages and decreases M2 polarization via LIF/p-Stat3 signaling and inhibits epithelial-mesenchymal transition (EMT) of HCC cells. Moreover, SM also affects other immune cell populations via macrophages to ameliorate the immunosuppressive microenvironment. Thus, these above-mentioned results demonstrate the potential use of SM for fighting HCC and shed new light on exploring SM as a potent small-molecule drug from traditional Chinese herb for the future HCC therapies (Fig. 2l).

## Supplementary Information

**Additional file 1**. Materials and Methods, Supplementary figures, Supplementary tables.

## Data Availability

All data relevant to this work are included in this paper and Additional file 1.

## Abbreviations

HCC: Hepatocellular carcinoma
SM: Solamargine
PDX: Patient-derived tumor xenograft
BA1: Bafilomycin A1
PPI: Protein–protein interaction
TAMs: Tumor associated macrophages
EMT: Epithelial-mesenchymal transition
