# Supplementary material for: Solamargine induces hepatocellular carcinoma cell apoptosis and autophagy via inhibiting LIF/miR-192-5p/CYR61/Akt signaling pathways and eliciting immunostimulatory tumor microenvironment
Source: J Hematol Oncol. 2022 Mar 21;15:32. doi: 10.1186/s13045-022-01248-w (PMC8935708; doi:10.1186/s13045-022-01248-w)
Supplement: Supplementary file 1 — Additional file 1. Materials and Methods, Supplementary figures, Supplementary tables. [file 13045_2022_1248_MOESM1_ESM.docx]

**Additional file 1 for**

**Solamargine induces hepatocellular carcinoma cell apoptosis and autophagy via inhibiting LIF/miR-192-5p/CYR61/Akt signaling pathways and eliciting immunostimulatory tumor microenvironment**

Shuangshuang Yin^1#^, Wenke Jin^2#^, Yuling Qiu^3#^, Leilei Fu^2^*, Tao Wang^1^*,

Haiyang Yu^1^*

Correspondence to: Leilei Fu ([leilei_fu@163.com](mailto:leilei_fu@163.com))

Tao Wang (wangtao@tjutcm.edu.cn)

Haiyang Yu (hyyu@tjutcm.edu.cn)

This file includes:

Materials and Methods

Supplementary figures S1 to S7

Supplementary tables S1 to S8

**MATERIALS AND METHODS**

1. **Cell culture** **and cell treatments**

Cell lines HepG2, HuH-7, SMMC-7721, THP-1, RAW 264.7 and H22 are purchased from the Cell Bank of Beijing Institute of Biochemistry and Cell Biology, Chinese Academy of Sciences (Beijing, China). The cells were cultured in Dulbecco’s modified Eagle’s medium (DMEM, Biological industry) plus 10% foetal bovine serum (FBS) and 1% penicillin G and streptomycin (Gibco, Grand Island, USA) at 37 °C in humidified air containing 5% CO_2_. The human monocytic cells (THP-1) were cultured in 1640 supplemented with 10% FBS. Solamargine (SM) with greater than 98% purity was obtained from Shanghai Yuanye Bio-Technology (Shanghai, China), dissolved in dimethyl sulfoxide (DMSO, Sigma-Aldrich) to make stock solutions and diluted to final concentrations in the culture medium. 4, 6-diamidino-2-phenylindole (DAPI) reagents were obtained from Sigma-Aldrich (St. Louis, MO, USA).

1. **Colony formation assay**

HCC cells were plated at 1 ×10^3^ per well in six-well plates, then removing the supernatant after co-cultured with Solamargine (SM) or DMSO (Con) for 24 h. After 10 days, cells were washed twice with cold PBS (Beijing Solarbio Science & Technology Co., Ltd., China), fixed with methanol (Tianjin Concord Technology Co., Ltd., China), and stained with 0.5% crystal violet (KeyGen Biotech, Nanjing, China). The number of colonies was counted under a microscope.

1. **MTT and apoptosis analysis**

Effects of SM on overall proliferation of HCC cells and macrophage cells were measured by using the MTT assay kit (Shanghai Yuanye Bio-Technology, China). Cells were seeded at 5 × 10^3^ cells/well into 96-well plates and incubated overnight, then treated with different concentrations of SM for another 24 h. Then, the MTT solution was added into each well, followed by 4 h of incubation, and absorbance was read at 550 nm by using a microplate reader. Apoptosis of HCC cells was examined by AnnexinV/propidium iodide Apoptosis Detection kit (BD Biosciences), samples were stained with 5 μl Annexin V-FITC for 10 min and with 5 μl propidium iodide (PI) for 3 min in the dark, and assessed by flow cytometry (Attune NxT, Invitrogen, CA, USA) under the guidance of the manufacturer’s instructions.

1. **Collection of conditioned medium (CM) and cancer cell invasion**

HepG2 and HuH-7 cells were plated in 6-well plates at a density of 5×10^5^ cells/well and the supernatants were collected as He-CM and Hu-CM after 24 h, respectively. RAW 264.7 and THP-1 cells pre-treated with SM for 24 h, then the medium was replaced with serum free DMEM and the cells were cultured for 24 h, the medium was collected as RSM-CM and TSM-CM. The indirect contact co-culture system was performed in 6-well plates with 8 μm polyethylene terephthalate membrane filters (Corning) separating the lower and upper chambers. M2 macrophage cells were pretreated or untreated with SM, then seeded into the upper chamber (1 × 10^5^) of a 6-well plate and co-cultured with HCC cells (2 × 10^5^) in the bottom chamber, cells were collected for analysis after 24 h.

1. **Immunofluorescence staining**

Cells were washed twice with cold PBS, and then fixed with 4% paraformaldehyde for 15 min and that permeabilization with 0.5% Triton X-100, after that these samples were blocked with 1% BSA containing 1% goat serum for 1 h. Then, samples incubated with primary antibodies overnight at 4 °C. Second day, samples followed by incubation with FITC-conjugated goat antimouse IgG or Alexa-Fluor 555-conjugated goat antirabbit IgG antibody for 1 h at room temperature. DAPI (Sigma-Aldrich) was used to stain nuclei of sphere cells. Cells were captured using an inverted fluorescent microscope (Carl Zeiss, Oberkochen, Germany) and quantified manually the acquired images with Image J software.

1. **Clinical samples**

Clinical samples of HCC patients were obtained from the First Affiliated Hospital of Zhejiang University. This study was approved by the Ethical Committee at the First Affiliated Hospital, Zhejiang University School of Medicine. All the subjects provided written informed consent. The details of the HCC patients were shown in Additional file 1: Table S1.

1. **Animal experiments**

Orthotopic mouse model: Tumor tissues were cut into about 1 mm^3^ pieces. One or two pieces were implanted in the left lobe of the liver in Kunming mice under anesthesia. The implanted tumors were allowed to grow 1 week. Then, the mice were divided into several groups at random and given indicated treatment (SM, 4 mg/kg, i.p., once every three days).

Patient-derived xenograft model: NSG mice were subcutaneously implanted with the early passage PDX tumor fragments. The implanted tumors were allowed to grow 1 week. Then, the mice were divided into two groups at random and given indicated treatment (SM, 4 mg/kg, i.v., once every three days). Patient information was shown in Additional file 1: Table S2.

Subcutaneous tumor model: 1 × 10^5^ of mouse H22 cells were injected subcutaneously into C57BL/6J mice. Secondly, macrophages in C57BL/6 mice were depleted by injection of clodronate-containing liposomes (Target Technology (Beijing) Co.,Ltd., an initiation dose, 200 μl, dose for preventing the repopulation of macrophages, 100 μl, once every five days). Then, the mice were divided into several groups at random and given indicated treatment (SM, 2 mg/kg, i.p., once every two days).

1. **Transmission electron microscopy**

After treatment, samples were harvested and washed in PBS (Beijing Solarbio Science & Technology Co., Ltd., China) and then fixed with 2.5% glutaraldehyde (Tianjin Concord Technology Co., Ltd., China), after 2 h, post-fixed in 1% osmium tetroxide. Then samples were dehydrated in acetone and embedded in epoxy resin at room temperature. Samples were cut into ultrathin sections (50 nm) using an ultramicro-microslicer, contrasted with uranyl acetate/lead citrate and the images of samples were obtained and analyzed with electron microscope Hitachi H-7650 (Hitachi, Tokyo, Japan).

1. **Western blot analysis**

For western blot analysis, total proteins were extracted and the protein concentrations were determined by using BCA kit (TK274303, Thermo Fisher Scientific, USA). Equal amounts of protein were added 4% SDS loading buffer and separated by sodium dodecyl sulfatepolyacrylamide gel electrophoresis, then transferred to polyvinylidene difluoride membranes (PVDF, Millipore, Billerica, MA). The membranes were incubated with corresponding primary and secondary antibodies. The detection was performed using ECL chemiluminescence kit (KF003, Affinity). The primary antibodies used were listed in Additional file 1: Table S3. Full scans of western blot assays were shown in Additional file 2: Fig. S1 to S8.

1. **Wound healing migration assay**

The inhibitory action on cell migration was further evaluated using a scratch wound healing assay. Tumor cells (1 × 10^6^) were inoculated into 6-well plates. The scratched cell “wound” was photographed at 0 and 24 h before and after treatments.

1. **Quantitative real time PCR assay**

In brief, total RNA was extracted by using Trizol reagent (TransGen Biotech, China) from HCC cells. RNA quantity and purity were determined using a NanoDrop 2000 (Thermo Scientific), and transcribed to cDNA using reverse transcription reagents (Fermentas China Co. Ltd., China). Relative mRNA and miRNA expression was detected by RT-qPCR using 7500 RT-PCR System (Applied Biosystems, Life Technologies). An SYBR PrimeScript RT-PCR Kit (Cowin Biotech) was used according to the manufacturer’s instructions. Actin (ACTB) and U6 were used as internal controls. The primer sequences used (GenePharma Co. Ltd., China) were listed in Additional file 1: Table S5, 8.

1. **Weighted correlation network analysis (WGCNA)**

We used the WGCNA package (version 1.70-3) based on R software (version 4.1) to identify differentially co-expressed gene modules obtained by our transcriptomics sequencing data expression matrix. Among them, for the phenotypic data of the samples, we only matched whether it is the Con or SM, which is represented by 0 and 1 in the matrix. To reduce the number of modules, highly correlated modules with a threshold of 0.02 are merged. Subsequently, we summarized the eigengenes of the module which were significantly associated with the SM for subsequent analysis.

1. **Plasmid, siRNAs and microRNA inhibitors transfection**

Human pcDNA-LIF, siLC3B and miR-192-5p inhibitor were purchased from GenePharma Co. Ltd., plasmids constructed by restriction-enzyme double digestion and ligation. pcDNA-LIF, siLC3B and miR-192-5p inhibitor transfection were performed with Lipofectamine 2000 (Invitrogen, USA) according to the supplier’s protocol. The sequences used were listed in Additional file 1: Table S6-7.

1. **Immunohistochemistry analysis**

In short, samples were fixed in 4% paraformaldehyde, and embedded in paraffin and then co-cultured with 1% BSA to block unspecific binding sites. Samples were incubated with primary antibody at 4 °C overnight and secondary antibody labeled with horseradish peroxidase at room temperature for 1 h. Finally, samples were counterstained with hematoxylin and visualized with 3, 30 -diaminobenzidine (DAB) tetrahydrochloride for 10 min. The samples were scanned by microscope (Olympus BX43, Olympus, Japan) and protein expressions were quantified by Image J. The primary antibodies used were listed in Additional file 1: Table S3.

1. **Macrophage polarisation *in vitro***

HCC cells were cultured in the DMEM-complete medium. After 24 h, the medium was recovered to prepare the tumor-conditioned medium. THP-1 cells were pretreated with PMA (200 nmol/L) for 24 h to induce differentiation. THP-1 and RAW 264.7 cells were treated with interleukin-4 (IL-4, 20 ng/ml) and interleukin-13 (IL-13, 20 ng/ml) or tumor-conditioned medium for 24 h to induce M2-like phenotype. The supernatant medium was removed, and then co-cultured with SM (2 μM) 24 h. Finally, cells were collected for qRT-PCR or western blot analysis.

1. ***In vitro* phagocytosis assay**

M2-like macrophages were treated with SM for 24 h, the culture medium was aspirated, and macrophages were co-cultured with HuH-7-GFP cells for 12 h. Then the FACS analysis to detect the phagocytosis of HuH-7-GFP cells by the macrophages. The percentage of GFP^+^ cells among F4/80^+^ macrophages was calculated as the ratio of phagocytosis.

1. **FACS analysis**

Tumor (spleen) single cell suspensions were stained with different antibodies avoiding light for 20-30 min at 4 °C, then washed two times with cold PBS. All sample analyzed by using Cytometer (Attune NxT, Thermo Fisher Scientific). The antibodies used were listed in Additional file 1: Table S4.

1. **Statistical analysis**

All experiments were performed at least three times and the data were analyzed by GraphPad Prism V.8.0. Student’s t-test and ANOVA analysis were performed between different groups. P < 0.05 was considered statistically significant. Actin (ACTB) and U6 as loading controls. Data were presented as means ± SD, ns means no significance, **p* < 0.05, ***p* < 0.01, ****p* < 0.001; ^#^*p* < 0.05, ^##^*p* < 0.01, ^###^*p* < 0.001.

**Fig. S1**


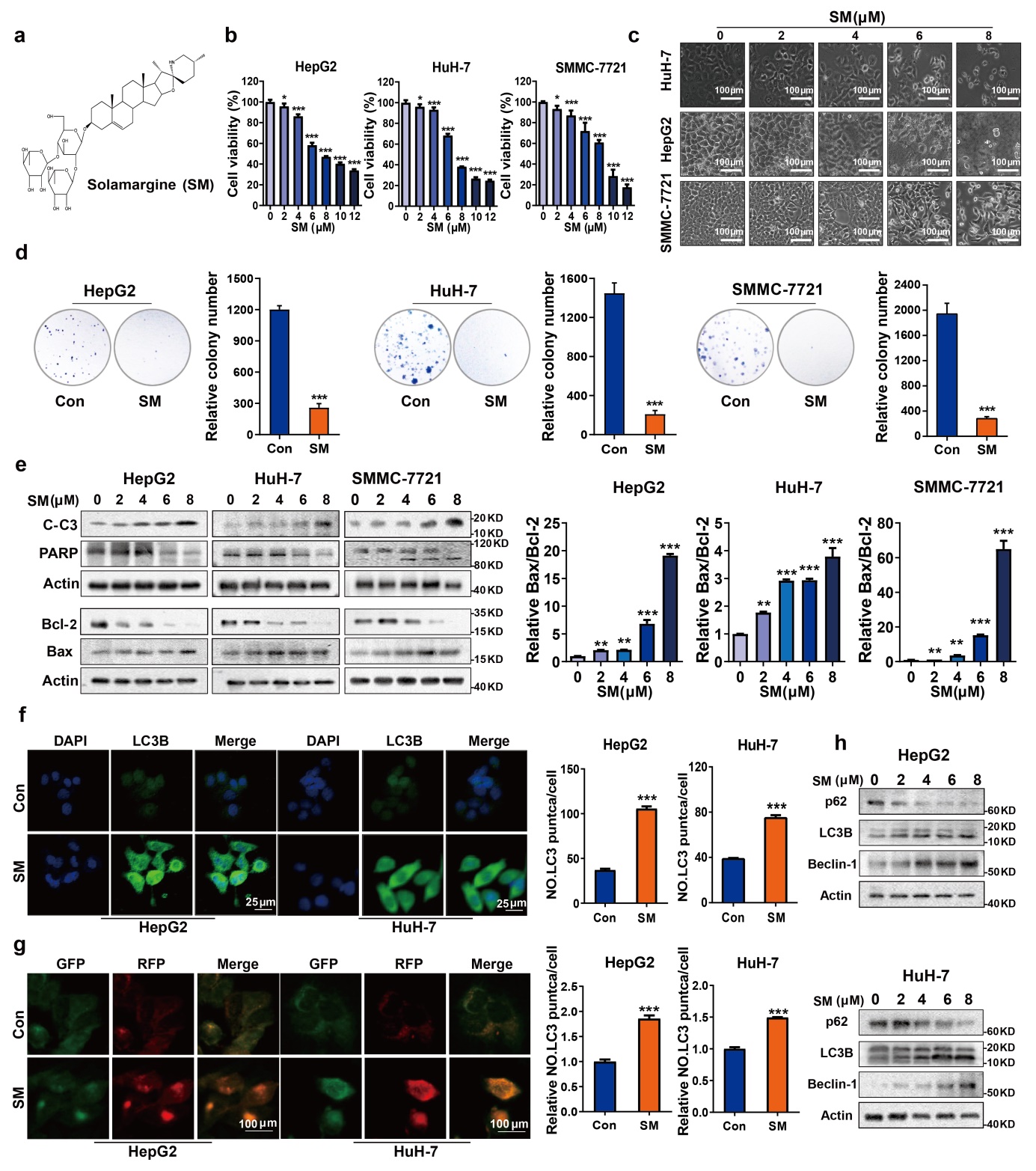


**Additional file 1: Fig. S1 Solamargine induces apoptosis and autophagy in HCC cells**

**a** The chemical structure of solamargine (SM). **b** Cell viability was measured by MTT assay. **c** The phase-contrast photomicrographs showed the morphology after SM treatment. **d** Colony formation assay of HCC cells treated with or without SM. Representative images and quantification of colonies were shown. **e** The expression of several cell apoptosis signal regulators, Cleaved-caspase 3 (C-C3), PARP, Bcl-2 and Bax, were examined by western blotting after treatment with SM (left). The quantification of BAX/Bcl-2 was shown (right). **f** Immunofluorescence analysis of the endogenous LC3B puncta in HCC cells. Representative images with quantification of LC3B intensity were shown. Scale bar, 25 µm. **g** Cells were transfected with GFP/mRFP-LC3 plasmid, after co-incubation with SM. Representative images and quantitative analysis of LC3 puncta were shown. Scale bar, 100 µm. **h** Western blot analysis of p62, LC3B and Beclin-1 expression in HepG2 and HuH-7 cells after treatment with the indicated concentrations of SM for 24 h. Actin was used as a loading control. Data were presented as means ± SD, ns means no significance, **p* < 0.05, ***p* < 0.01, ****p* < 0.001.

**Fig. S2**


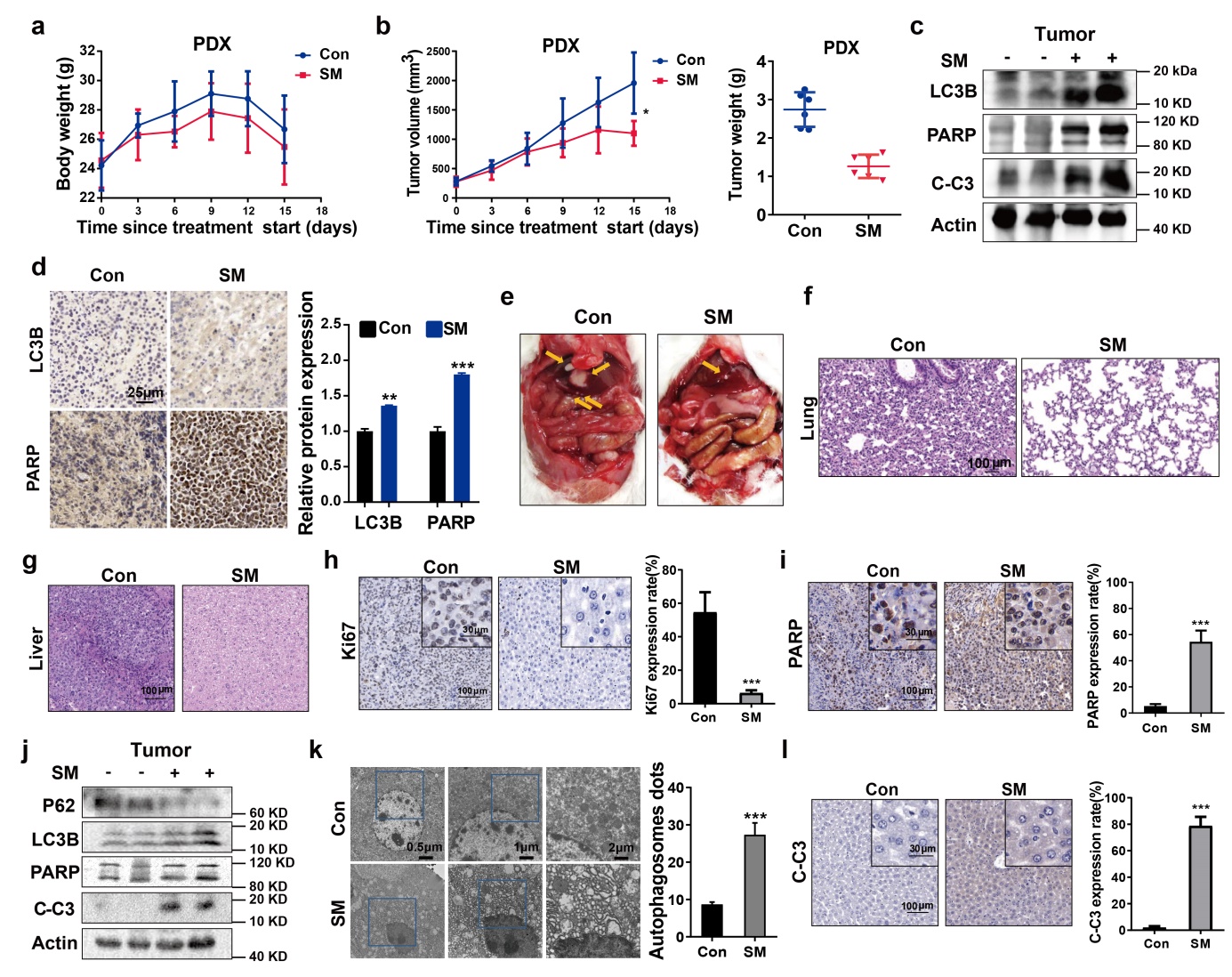


**Additional file 1: Fig. S2 Solamargine inhibits tumor growth *in vivo***

**a** Body weight of patient-derived tumor xenografts mice. **b** The tumor volume (left) the final tumor weight (right) of patient-derived tumor xenografts mice (n = 10 for each group). **c** The expression of LC3B, PARP and C-C3 in tissue of patient-derived tumor xenografts mice were determined by western blot assay. **d** Representative micrographs of LC3B and PARP expression in tissue of patient-derived tumor xenografts mice, as analyzed by IHC. Quantitative analysis of the percentage of positive ratios was shown. Scale bars, 25 μm. **e** Representative images of orthotopic HCC tumors after after SM treatment. **f** Histopathology of the lung tissues dissected from orthotopic HCC mice. Representative images were shown. **g** Histopathology of the liver tissues dissected from orthotopic HCC mice. Representative images were shown. **h-i and l** The expressions of Ki67, PARP and C-C3 in liver tissues dissected from orthotopic HCC mice were examined by IHC. Representative images and quantitative analysis of the percentage of positive ratios were shown. **j** The expressions of p62, LC3B, PARP and C-C3 were determined in tumor tissue of the orthotopic HCC mice by western blot assays. **k** Autophagy was measured by transmission electron microscopy in tumor tissues of the orthotopic HCC mice. Actin was used as a loading control. Data were presented as means ± SD, ns means no significance, **p* < 0.05, ***p* < 0.01, ****p* < 0.001.

**Fig. S3**

**
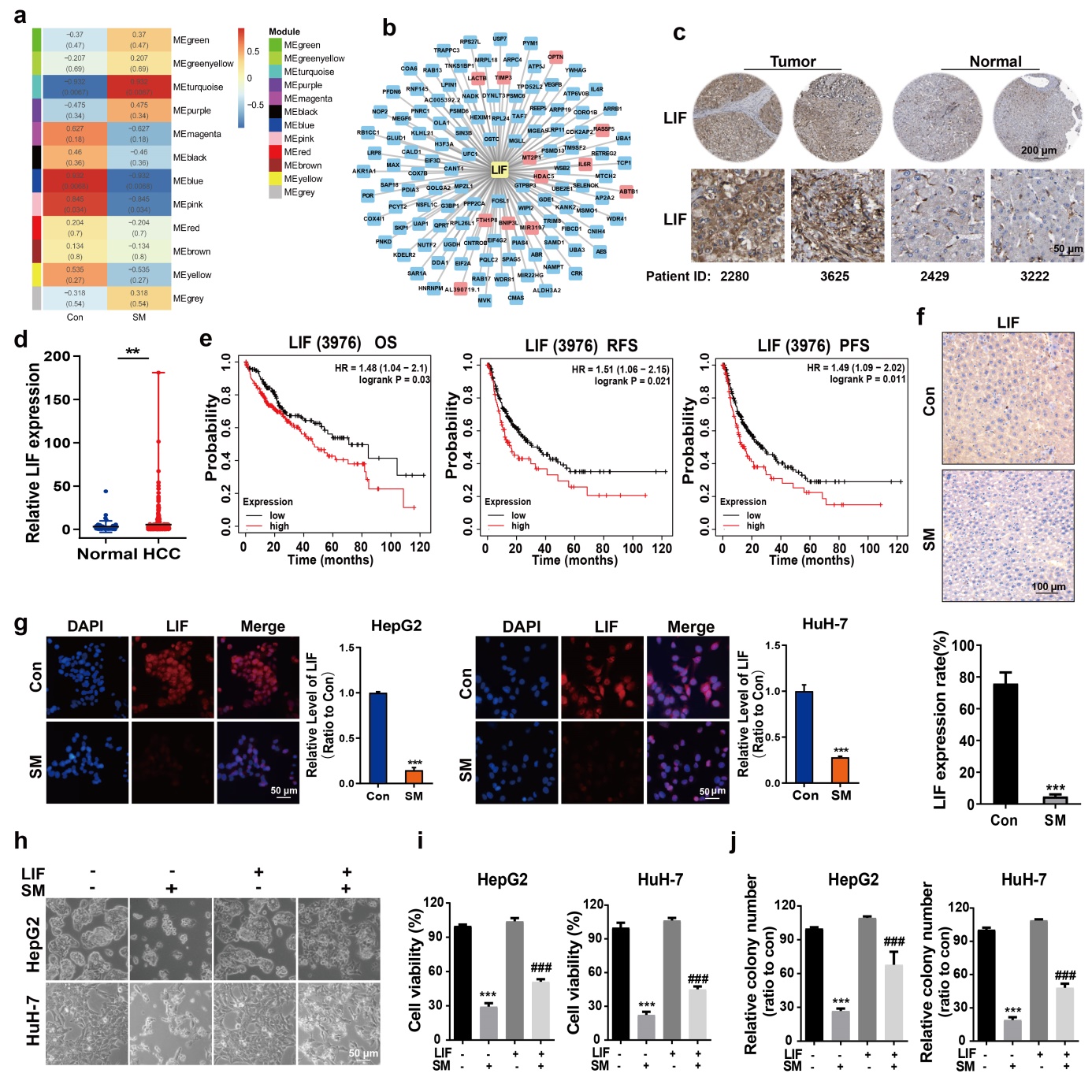
**

**Additional file 1: Fig. S3 Solamargine inhibits HCC growth via blocking LIF**

**a** The results of differentially expressed genes were displayed by WGCNA analysis. **b** the network of transcriptomics sequencing data were constructed by co-expression data of the turquoise module. **c** The expression of LIF in HCC patient and normal tissues obtained from HPA. **d** The expression of LIF in Human HCC specimens and normal tissues was analyzed by TCGA. **e** The prognostic survival of HCC patients with LIF expression. **f** The expression of LIF in tumor tissues of orthotropic HCC mice (up). Quantification of positive LIF was shown (bottom). **g** Immunofluorescence analysis of LIF in HCC cells treated with or without SM. Representative images and quantification of LIF intensity were shown. Scale bar, 50 µm. **h** The phase-contrast photomicrographs showed the morphology after SM combined with LIF-plasmid or SM alone treatment. **i** Cell viability of HCC cells was ascertained after SM plus LIF-plasmid or SM alone treatment. **j** Quantification of Colony formation assay of HCC cells treated with SM combined LIF-plasmid or SM alone. Actin was used as a loading control. Data were presented as means ± SD, ns means no significance, **p* < 0.05, ***p* < 0.01, ****p* < 0.001; ^#^*p* < 0.05, ^##^*p* < 0.01, ^###^*p* < 0.001.

**Fig. S4**


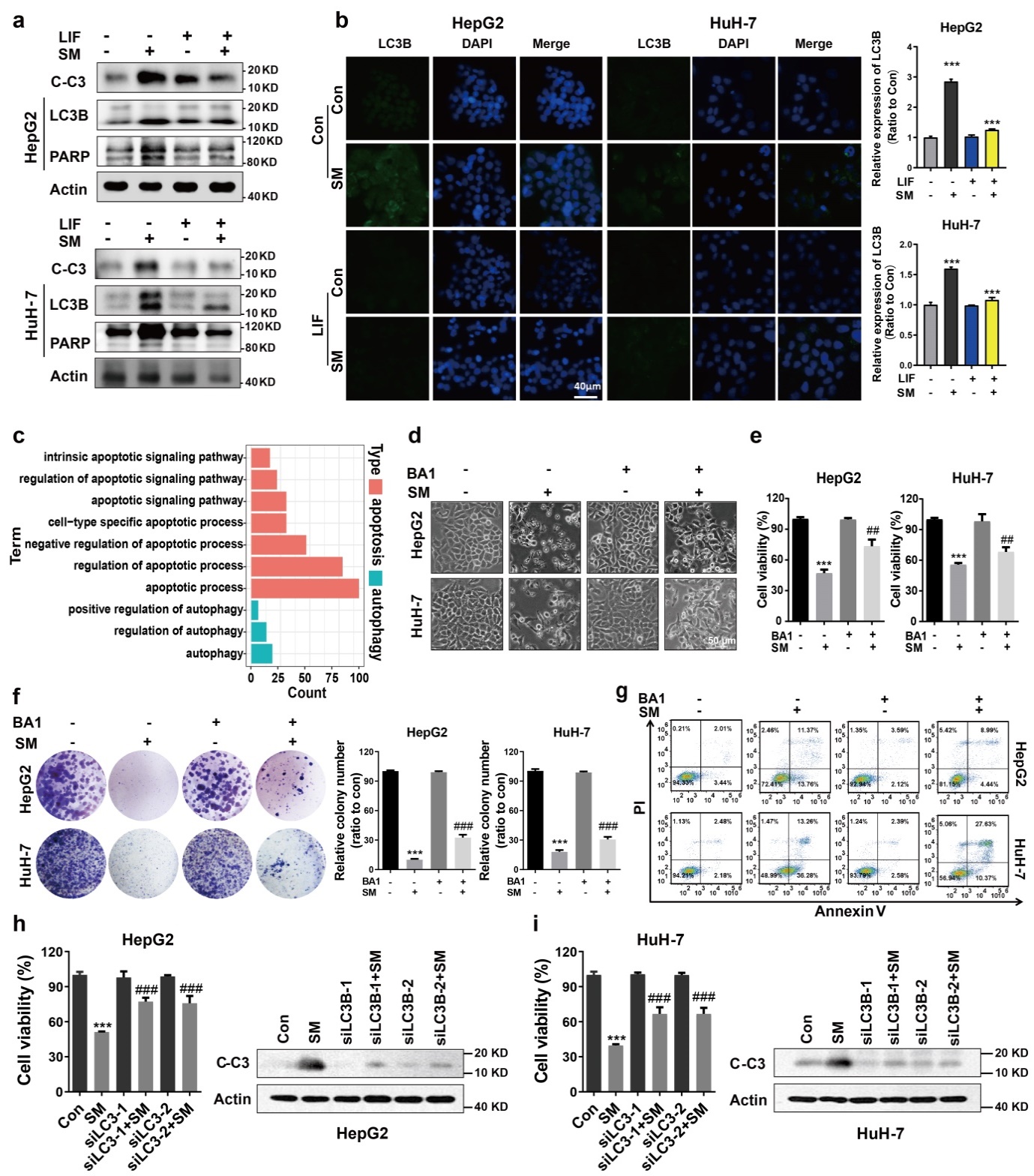


**Additional file 1: Fig. S4 Inhibition of autophagy alleviates HCC cell death induced by Solamargine**

**a** The expressions of C-C3, LC3B, PARP were determined after SM plus LIF-plasmid or SM alone treatment in HCC cells. **b** Immunofluorescence analysis of LC3B in HCC cells treated with SM plus LIF-plasmid or SM alone. Representative images and quantification of LC3B intensity were shown. Scale bar, 40 µm. **c** KEGG analysis of apoptosis/autophagy-related genes with significant differences expression in HepG2 cells treated with or without SM. **d** The phase-contrast photomicrographs showed the morphology after SM plus BA1 or SM alone treatment. **e** Cell viability of HCC cells was determined after SM plus BA1 or SM alone treatment. **f** Colony formation assay of HCC cells treated with SM combined BA1 or SM alone. Representative images and quantification of colonies were shown. **g** Representative results of Annexin V-FITC/PI staining of HCC cells treated with SM combined BA1 or SM alone. **h and i** Cell viability of HCC cells was detected by MTT assay (left). HCC cells were treated with SM plus siLC3B or SM alone, C-C3 expression was detected by western blot assay (right). Actin was used as a loading control. Data were presented as means ± SD, ns means no significance, **p* < 0.05, ***p* < 0.01, ****p* < 0.001; ^#^*p* < 0.05, ^##^*p* < 0.01, ^###^*p* < 0.001.

**Fig. S5**


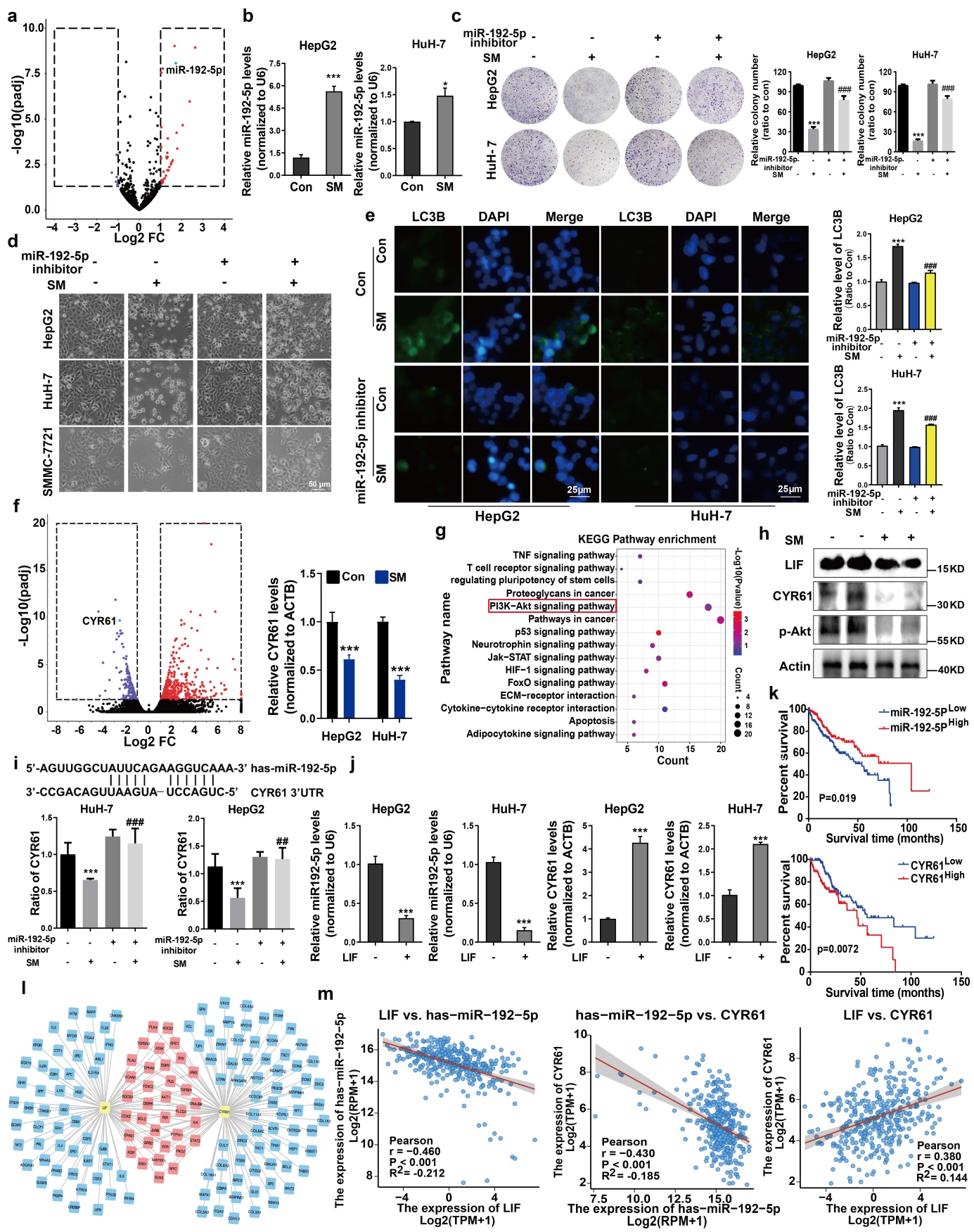


**Additional file 1: Fig. S5 Solamargine exerts anti-HCC potential via LIF/miR-192-5p/CYR61/Akt axis**

**a** Volcano plot of differentially expressed miRNAs with significant differences expression in HepG2 cells treated with or without SM. **b** The expression of miR-192-5p was tested by qRT-PCR analysis in HCC cells. **c** Colony formation assay of HCC cells treated with SM combined miR-192-5p inhibitor or SM alone. Representative images and quantification of colonies were shown. **d** The phase-contrast photomicrographs showed the morphology after SM plus miR-192-5p inhibitor or SM alone treatment. **e** Immunofluorescence analysis of LC3B in HCC cells treated with SM plus miR-192-5p inhibitor or SM alone. Representative images with quantification of LC3B intensity were shown. **f** Volcano plot of differentially expressed genes with significant differences expression in HCC cells treated with or without SM (left). The expression of CYR61 was tested by qRT-PCR analysis in HCC cells treated with or without SM. (right). **g** KEGG analysis of differentially expressed genes with significant differences expression in HepG2 cells treated with or without SM. **h** The expressions of LIF, CYR61 and p-Akt were determined in tumor tissue of patient-derived tumor xenografts mice by western blot assay. **i** miR-192-5p binding to CYR61 in UTR site was predicted by TargetScan database (up). The mRNA level of CYR61 was tested by qRT-PCR analysis in HCC cells treated with SM plus miR-192-5p inhibitor or SM alone (bottom). **j** miR-192-5p and CYR61 expression were tested by qRT-PCR analysis in HCC cells treated with LIF-plasmid. **k** The prognostic survival of HCC patients with miR-192-5p or CYR61 expression. **l** PPI of CYR61 has a significant overlap with the PPI of LIF constructed by cytoscape. **m** The relevance between LIF and relative expression of miR-192-5p in clinical HCC samples (left). The relevance between miR-192-5p and relative expression of CYR61 in clinical HCC samples (middle). The relevance between LIF and relative expression of CYR61 in clinical HCC samples (right). Actin (ACTB) and U6 were used as loading controls. Data were presented as means ± SD, ns means no significance, **p* < 0.05, ***p* < 0.01, ****p* < 0.001; ^#^*p* < 0.05, ^##^*p* < 0.01, ^###^*p* < 0.001.

**Fig. S6**


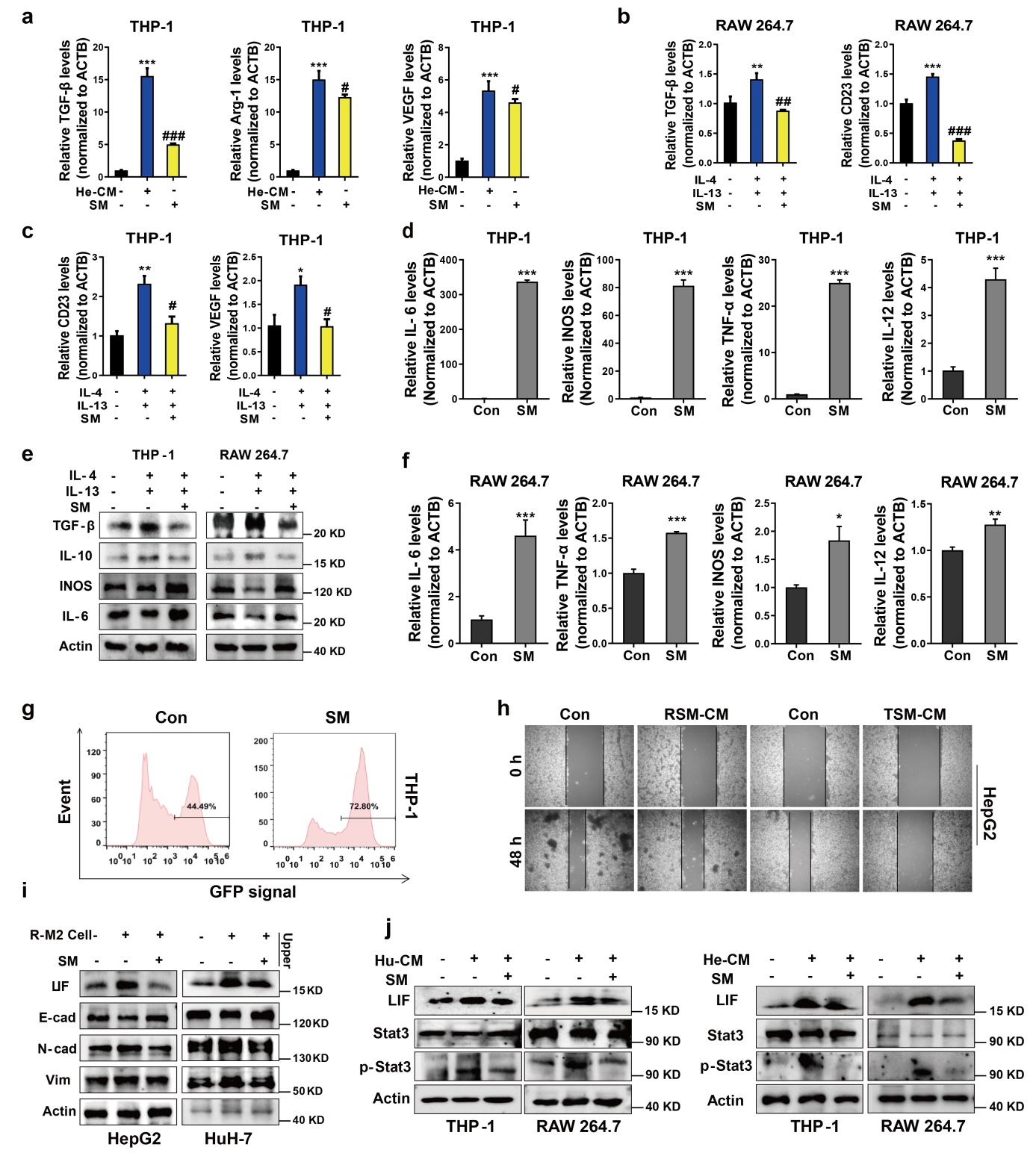


**Additional file 1: Fig. S6 Solamargine repolarizes M2 macrophages toward M1 phenotype**

**a-c** Macrophages were co-cultured with tumor-conditioned medium (TCM) or IL-4 and IL-13 for 24 h to induce M2-like macrophage, then treatment with SM for 24 h, the expressions of M2 associated genes were measured by qRT-PCR assay. **d and f** Macrophages were co-cultured with SM for 24 h, the expressions of M1 associated genes (INOS, IL-6,TNF-α, IL-12) were measured by qRT-PCR assay. **e** Macrophages were co-cultured with IL-4 and IL-13 for 24 h to induce M2-like macrophage, then treated with SM for 24 h, the expressions of M2 associated genes (TGF-β, IL-10) and M1 associated genes (INOS, IL-6) were measured. **g** The signal intensity of GFP in the F4/80 positive macrophage was demonstrated by flow cytometry. **h** HCC cells were treated with or without RSM-CM or TSM-CM. The scratch assay was used to measure migration capabilities of HCC cells. Representative images were shown. **i** The expression of LIF, E-cad, N-cad and Vim were determined after M2 macrophage were pretreated with SM and placed in the upper chamber to test the invasion ability of HCC cells in the lower chamber. j The expression of LIF, p-Stat3 and total Stat3 were examined by western blotting after co-cultured with TCM with or without SM. Actin (ACTB) was used as a loading control. Data were presented as means ± SD, ns means no significance, **p* < 0.05, ***p* < 0.01, ****p* < 0.001; ^#^*p* < 0.05, ^##^*p* < 0.01, ^###^*p* < 0.001.

**Fig. S7**


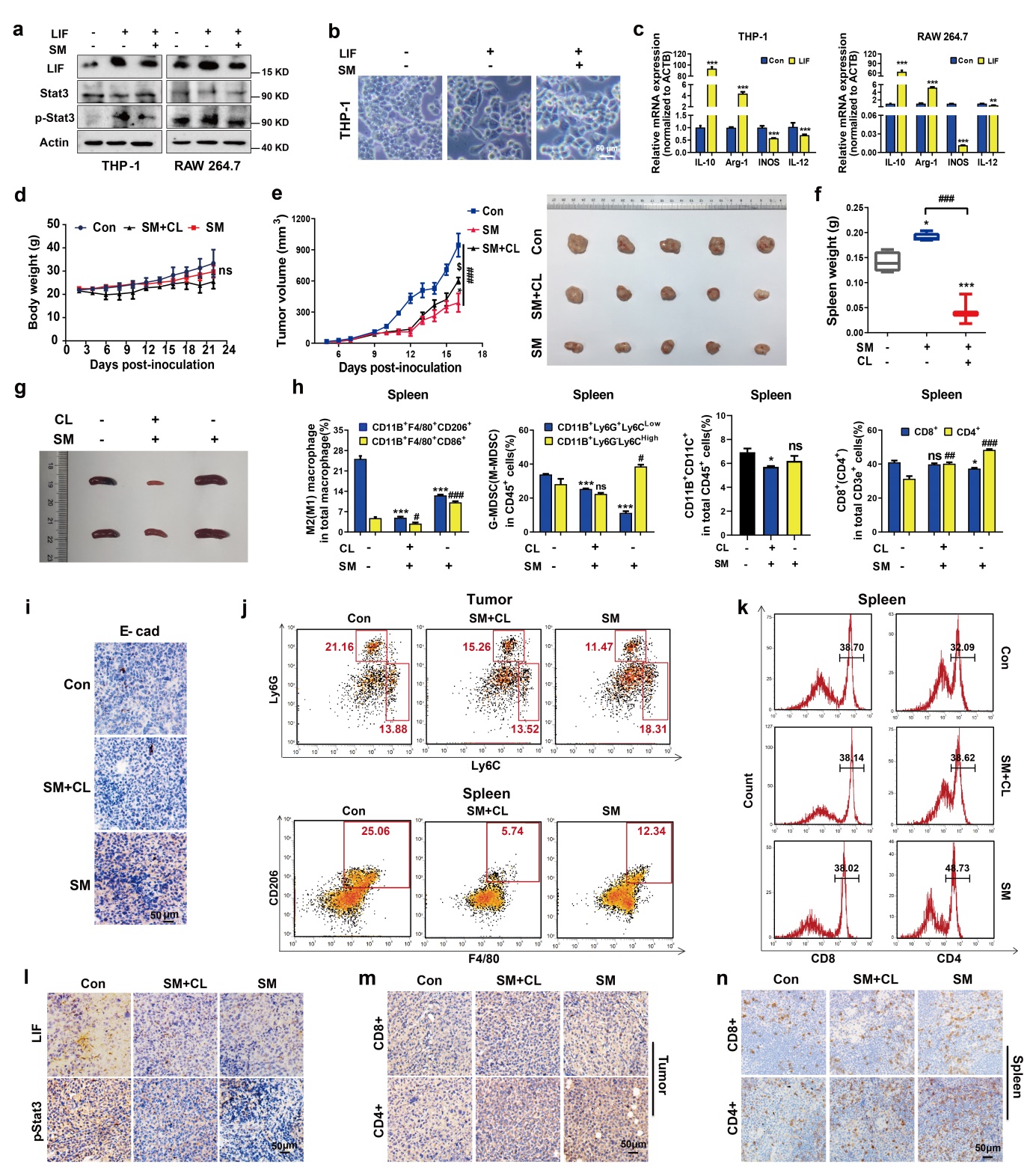


**Additional file 1: Fig. S7 Macrophages are required for SM to modulate the tumor microenvironment of HCC**

**a** The expression of LIF, p-Stat3 and total Stat3 were examined by western blotting after co-cultured with LIF-plasmid with or without SM. **b** The phase-contrast photomicrographs showed the morphology after SM plus LIF-plasmid or SM alone treatment. **c** The expressions of M2 associated genes and M1 associated genes in macrophages after transfection of LIF plasmid were measured by qRT-PCR assay. **d** Body weight of H22 subcutaneous tumor mice. **e** The tumor volume (left) and representative photographs of the final tumor tissue (right) of H22 subcutaneous tumor mice (n = 9). **f** The final spleen weight of H22 subcutaneous tumor mice. **g** The representative photographs of final spleen weight of H22 subcutaneous tumor mice. **h, j and k** Flow cytometry analysis of tumor-associated macrophages, DC cells (CD11B^+^CD11C^+^), MDSCs (G-MDSCs, CD11B^+^Ly6G^+^Ly6C^Low^; M-MDSCs, CD11B^+^Ly6C^High^Ly6G^-^) and infiltrating T cells (CD4^+^ T, CD3e^+^CD4^+^; CD8^+^ T, CD3e^+^CD8^+^) in endpoint tumors (spleens). Quantitative analysis and representative photographs of the positive ratios were shown. **i and l** E-cad, p-Stat3 and LIF were determined in tumor tissue of H22 subcutaneous mice by Immunohistochemical analysis. **m and n** CD4 and CD8 were determined in tumor/spleen tissues of H22 subcutaneous tumor mice by Immunohistochemical analysis. Actin (ACTB) was used as a loading control. Data were presented as means ± SD, ns means no significance, **p* < 0.05, ***p* < 0.01, ****p* < 0.001; ^#^*p* < 0.05, ^##^*p* < 0.01, ^###^*p* < 0.001.

**Additional file 1: Table S1. Patient information (Case1-8)**

| **Case No.** | | **Gender** | **Tumor Grade** | | **Tumor size (cm)** | **carcinoembryonic antigen (CEA) (ng/ml)** | **Alpha fetoprotein (AFP)** |
| --- | --- | --- | --- | --- | --- | --- | --- |
| #1 | Male | | Ⅰ-Ⅱ | 3 ×2 | | 4.4 | 48.1 |
| #2 | Male | | NA | 3.5 ×3 ×3 | | 2.4 | 3.2 |
| #3 | Male | | Ⅱ | 2.5 ×2.5 | | 1.8 | 2.5 |
| #4 | Male | | Ⅱ-Ⅲ | 5.5 ×5 ×4.5 | | NA | NA |
| #5 | Female | | Ⅱ | 2 ×1 | | 1.1 | 234.6 |
| #6 | Male | | Ⅱ-Ⅲ | 3 ×1.5 | | 1.4 | 6.8 |
| #7 | Male | | Ⅱ-Ⅲ | 5 ×4 | | 2.9 | 87.7 |
| #8 | Male | | Ⅱ-Ⅲ | 1.5 ×1.4 | | 1.6 | 2.4 |

**Additional file 1: Table S2. Patient information (PDX model)**

| Gender | Female |
| --- | --- |
| Pathological diagnosis | Hepatocellular carcinoma |
| Tumor size (cm) | 1.7×1.5×1.4 |
| Tumor stage | Ⅱ |

**Additional file 1: Table S3. Antibodies**

| **Antibodies** | **Source** | **Identifier** | **Antibody dilutions(for WB)** | **Antibody dilutions(for IHC)** | **Antibody dilutions(for IHC)** |
| --- | --- | --- | --- | --- | --- |
| p-Akt (S473) | CST | 4060 | 1:1000 | 1:250 | - |
| Akt (pan) | CST | 4691 | 1:1200 | - | - |
| C-C3 | CST | 9661 | 1:1000 | 1:200 | - |
| PARP | CST | 9542 | 1:1000 | 1:200 | - |
| Bcl-2 | CST | 15071 | 1:1000 | - | - |
| Bax | CST | 14796 | 1:1000 | - | - |
| p62 | CST | 8025 | 1:1000 | - | - |
| LC3B | abcam | ab48394 | 1:1200 | 1:200 | 1:200 |
| Beclin-1 | CST | 3495 | 1:1200 | - | - |
| LIF | abcam | ab113262 | 1:1000 | 1:200 | 1:200 |
| CYR61 | proteintech | 26689-1-AP | 1:800 | 1:250 | - |
| TGF-β | abcam | ab215715 | 1:1200 | - | - |
| IL-10 | CST | 12163 | 1:1200 | - | - |
| INOS | abcam | ab178945 | 1:1200 | - | - |
| IL-6 | abcam | ab259341 | 1:1200 | - | - |
| Vim | servicebio | GB11192 | 1:1000 | - | - |
| E-cad | CST | 3195 | 1:1200 | 1:200 | - |
| N-cad | CST | 84117 | 1:1200 | - | - |
| COX-2 | proteintech | 27308-1-AP | 1:1200 | - | - |
| TNF-α | proteintech | 17590-1-AP | 1:1000 | - | - |
| Ki67 | abcam | ab15580 | - | 1:250 | - |
| Actin | abcam | ab8227 | 1:1200 | - | - |
| CD4 | abcam | ab183685 | - | 1:200 | - |
| CD8 | bioss | bs-0648R | - | 1:150 | - |
| p-Stat3 (Tyr705) | CST | 9145 | 1:1000 | 1:200 | - |
| Stat3 | CST | 30835 | 1:1000 | - | - |
| F4/80 | Affinity | DF2789 | - | - | 1:150 |
| CD206 | Affinity | DF4149 | - | - | 1:150 |

**Additional file 1: Table S4. Antibodies for FACS**

| **Antibodies** | **Source** | **Identifier** |
| --- | --- | --- |
| CD45 | Thermo fisher | 69-0451-82 |
| CD3 | Thermo fisher | 15-0031-82 |
| CD4 | Thermo fisher | 56-0041-82 |
| CD8 | Thermo fisher | MCD0817 |
| Ly6G | biolegend | 127608 |
| Ly6C | biolegend | 128032 |
| CD11B | biolegend | 101257 |
| F4/80 | Thermo fisher | 25-4801-82 |
| CD206 | biolegend | 141710 |
| CD86 | Thermo fisher | 53-2061-82 |
| CD11C | Thermo fisher | 45-0114-80 |
| Fixable Viability Dye | Thermo fisher | 65-0864-14 |

**Additional file 1: Table S5. Primer sequences for qRT-PCR**

| **Gene** | **Forward Primer (5’-3’)** | **Reverse Primer (5’-3’)** |
| --- | --- | --- |
| Actin | CTGGAACGGTGAAGGTGACA | AAGGGACTTCCTGTAACAATGCA |
| CYR61 | GGTCAAAGTTACCGGGCAGT | GGAGGCATCGAATCCCAGC |
| miR-192-5p | ATACAGGATAACGATTGACG | GCTCTAGAGATCACATAG |
| U6 | AATCCTTCATTCCACCGG | AACGCTTCACGAATTTGCGT |

**Additional file 1: Table S6. siRNA sequences in RNA interference**

| **Gene** | **siRNA sense (5’-3’)** | **siRNA antisense (5’-3’)** |
| --- | --- | --- |
| siLC3B-1 UAAGUCGGACAUCUUCUACdTdT UUGAAGGUCUUCUCCGACGdTdT | UUCUCCGAACGUGUCACGUdTdT | ACGUGACACGUUCGGAGAAdTdT |
| siLC3B-2 | GUAGAAGAUGUCCGACUUAdTdT | CGUCGGAGAAGACCUUCAAdTdT |
| NC | UUCUCCGAACGUGUCACGUdTdT | ACGUGACACGUUCGGAGAAdTdT |

**Additional file 1: Table S7. Plasmid-LIF and microRNA inhibitors**

| **Gene** | **sequences** |
| --- | --- |
| pcDNA-LIF | 5’-CGCAAATGGGCGGTAGGCGTG-3’ |
| miR-192-5p inhibitor | 5’-GGCUGUCAAUUCAUAGGUCAG-3’ |

**Additional file 1: Table S8. Primer sequences for qRT-PCR**

| **Gene (Human)** | **Forward Primer**  **(5’-3’)** | **Reverse Primer**  **(5’-3’)** |
| --- | --- | --- |
| TGF-β | CGCTGCCCATCGTGTACTA | CCAATGACACAGAGATCCGC |
| Arg-1 | ACTTAAAGZZCAAGAGTGTGATGTG | CATGGCCAGAGATGCTTCCA |
| IL-10 | TACGGCGCTGTCATCGATTT | TAGAGTCGCCACCCTGATGT |
| CD23 | TCCTGCTTAAACCTCTGTCTCTG | AGCTCTGGTTAGTGGAGTTTGG |
| VEGF | TCCGGGCTCGGTGATTTA | GACTCCGGCGGAAGCAT |
| IL-6 | TCAATATTAGAGTCTCAACCCCCA | GAGAAGGCAACTGGACCGAA |
| INOS | AAGCAGCAGAATGAGTCCCC | CCTGGGTCCTCTGGTCAAAC |
| TNF-α | ACCCACGGCTCCACCCTCTC | CCCTCTGGGGGCCGATCACT |
| IL-12 | ACTCACCTCTTCAGAACGAATTG | ACAGGGCCATCATAAAAGAGGT |
| Actin | CTGGAACGGTGAAGGTGACA | AAGGGACTTCCTGTAACAATGCA |

| **Gene**  **(Mouse)** | **Forward Primer**  **(5’-3’)** | **Reverse Primer**  **(5’-3’)** |
| --- | --- | --- |
| TNF-α | ACCCACGGCTCCACCCTCTC | CCCTCTGGGGGCCGATCACT |
| IL-12 | CAATCACGCTACCTCCTCTTTT | CAGCAGTGCAGGAATAATGTTTC |
| TGF-β | CTTCAATACGTCAGACATTCGGG | GTAACGCCAGGAATTGTTGCTA |
| Arg-1 | TTGGGTGGATGCTCACACTG | TTGCCCATGCAGATTCCC |
| IL-10 | CTTACTGACTGGCATGAGGATCA | GCAGCTCTAGGAGCATGTGG |
| CD23 | CTCTCCCAGAACCTGAACAGACTC | AGCCCTTGCCAAAATAGTAGCAC |
| VEGF | CCGCAGACGTGTAAATGTTCCT T | TTCCGGTGAGAGGTCTGGTTC |
| IL-6 | CTGCAAGAGACTTCCATCCAG | AGTGGTATAGACAGGTCTGTTGG |
| INOS | CAGCTGGGCTGTACAAACCTT | CATTGGAAGTGAAGCGTTTCG |
| Actin | ACAACCTTCTTGCAGCTCCTC | CTGACCCATACCCACCATCAC |
